# Supplementary material for: Tremor activity inhibited by well-drained conditions above a megathrust
Source: Nat Commun. 2016 Dec 19;7:13863. doi: 10.1038/ncomms13863 (PMC5187422; doi:10.1038/ncomms13863)
Supplement: Supplementary Information — Supplementary Figures 1-7 and Supplementary References. [file ncomms13863-s1.pdf]

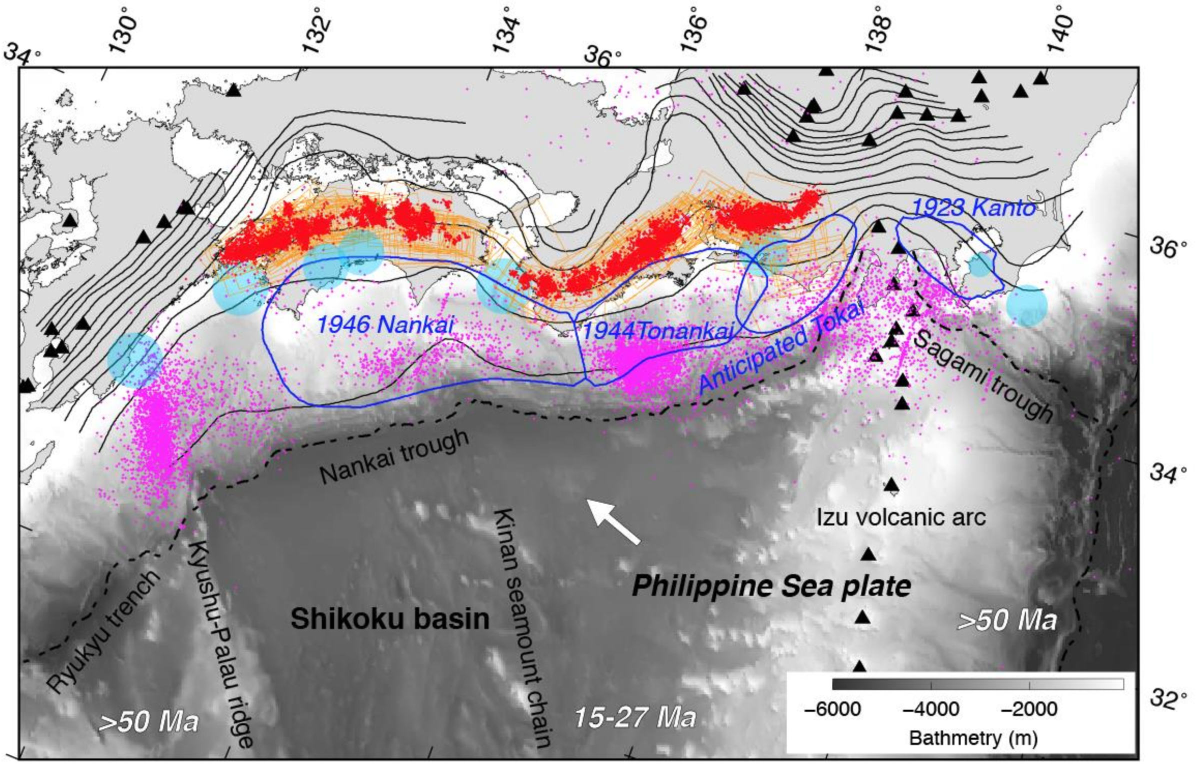

Supplementary Figure 1. Tectonic setting of southwestern Japan with bathymetry indicated by the grey scale shading. Various types of shear slips are present in the study area: very low-frequency earthquakes[1] (purple dots), long-term SSEs[2–9] (blue circles), short-term SSEs[10] (orange squares), and LFEs determined by the Japan Meteorological Agency (red dots). Source areas of past and anticipated megathrust earthquakes are denoted by the blue lines. The upper surface of the Philippine Sea plate[11,12] and active volcanoes are shown by the black lines and triangles, respectively.

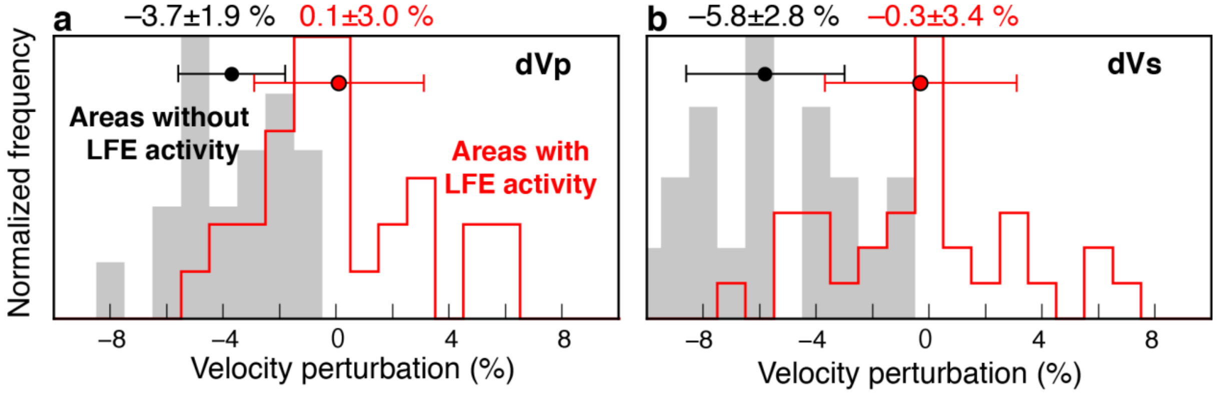

Supplementary Figure 2. Normalized frequency distribution of **(a)** P-wave and **(b)** S-wave velocity perturbations along the LFE band with LFE activity (red histogram) and without LFE activity (grey histogram). The P-wave and S-wave perturbations shown in Figs. 2(a) and 2(b) are used for this calculation. Mean values and one-sigma uncertainties are shown by the circles and bars, respectively.

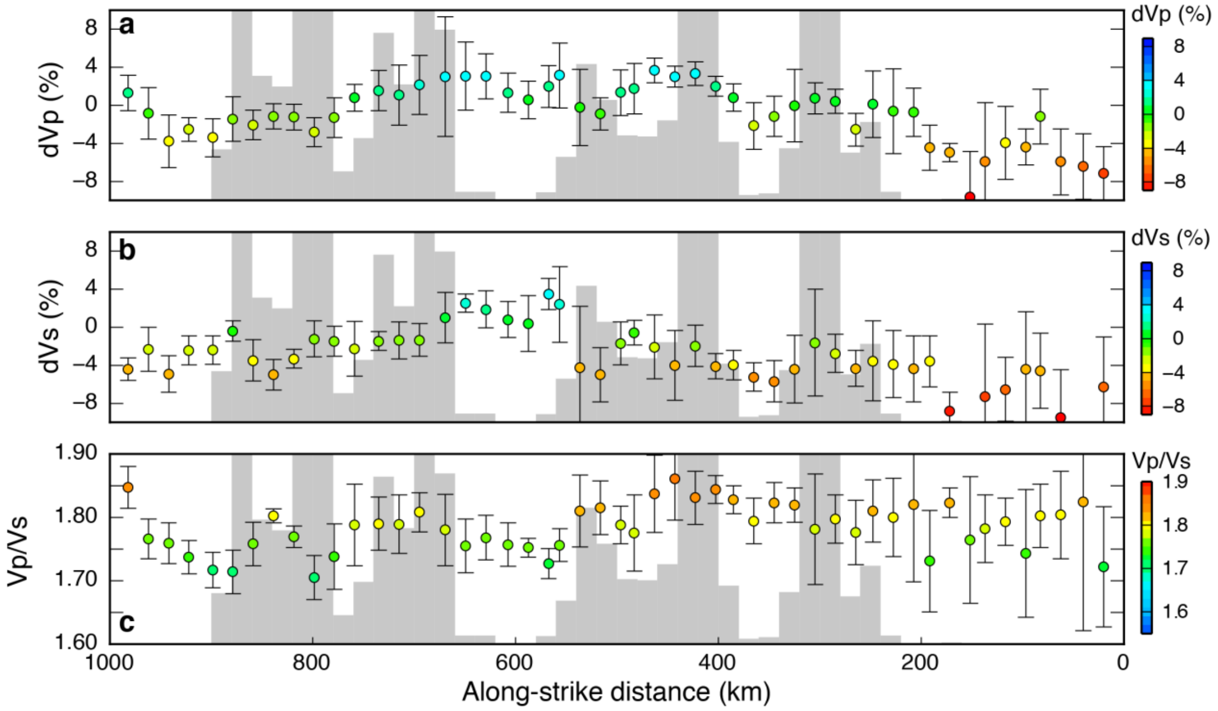

Supplementary Figure 3. Seismic velocities in the subducting crust compared to tremor activity. Along-strike variations in **(a)** P-wave and **(b)** S-wave velocity perturbations and **(c)** Vp/Vs values averaged over the layer 1–4 km below the upper surface of the Philippine Sea slab beneath the LFE band in shown in Fig. 1(a). The vertical bars adjacent to each data point in the panels denote one-sigma uncertainties. The grey histogram in each panel denotes the number of LFEs (the maximum frequency of 1000).

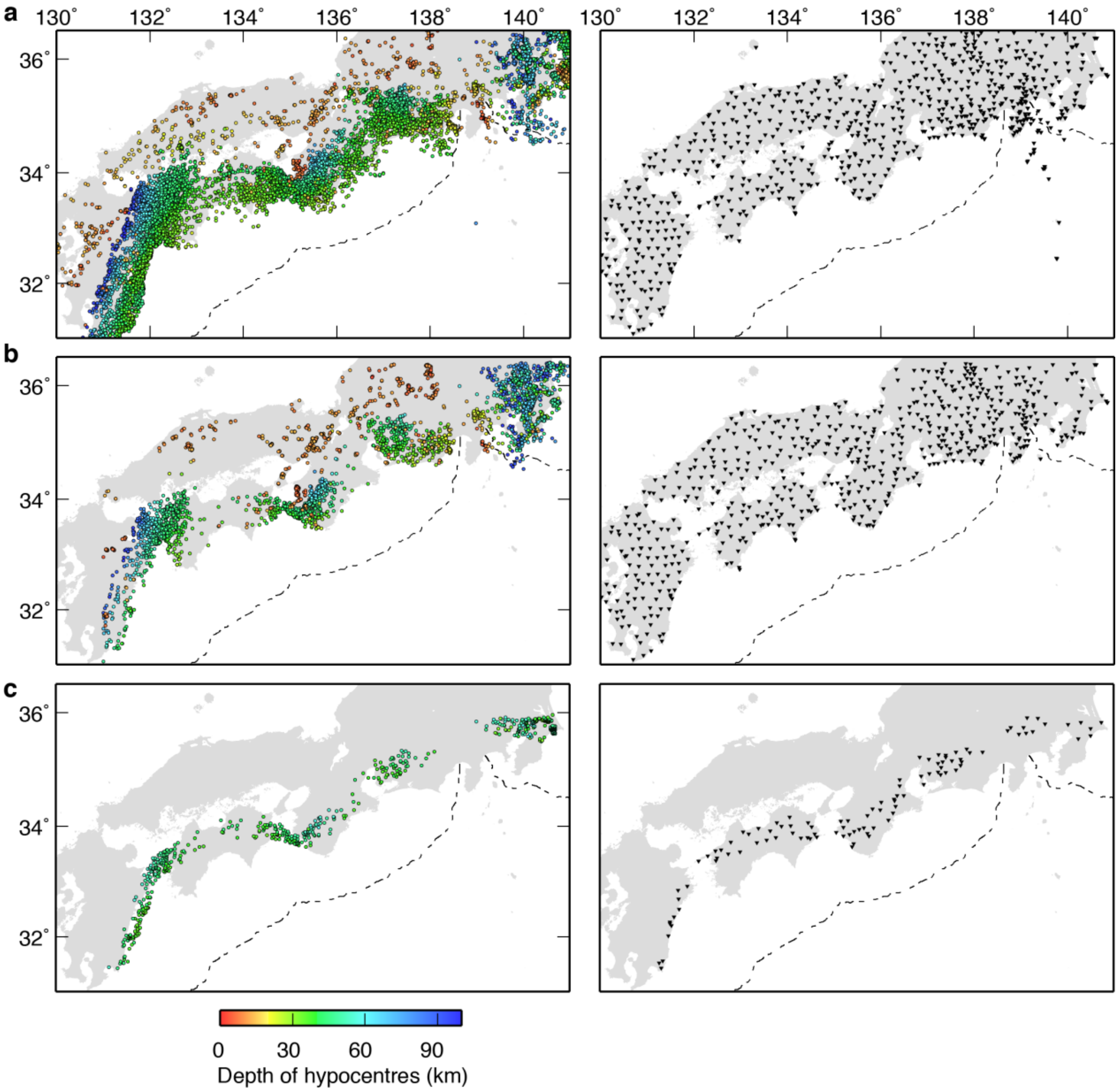

Supplementary Figure 4. Distribution of hypocentres (left) and stations (right) for **(a)** travel-time tomography, **(b)** attenuation tomography, and **(c)** shear-wave splitting analysis.

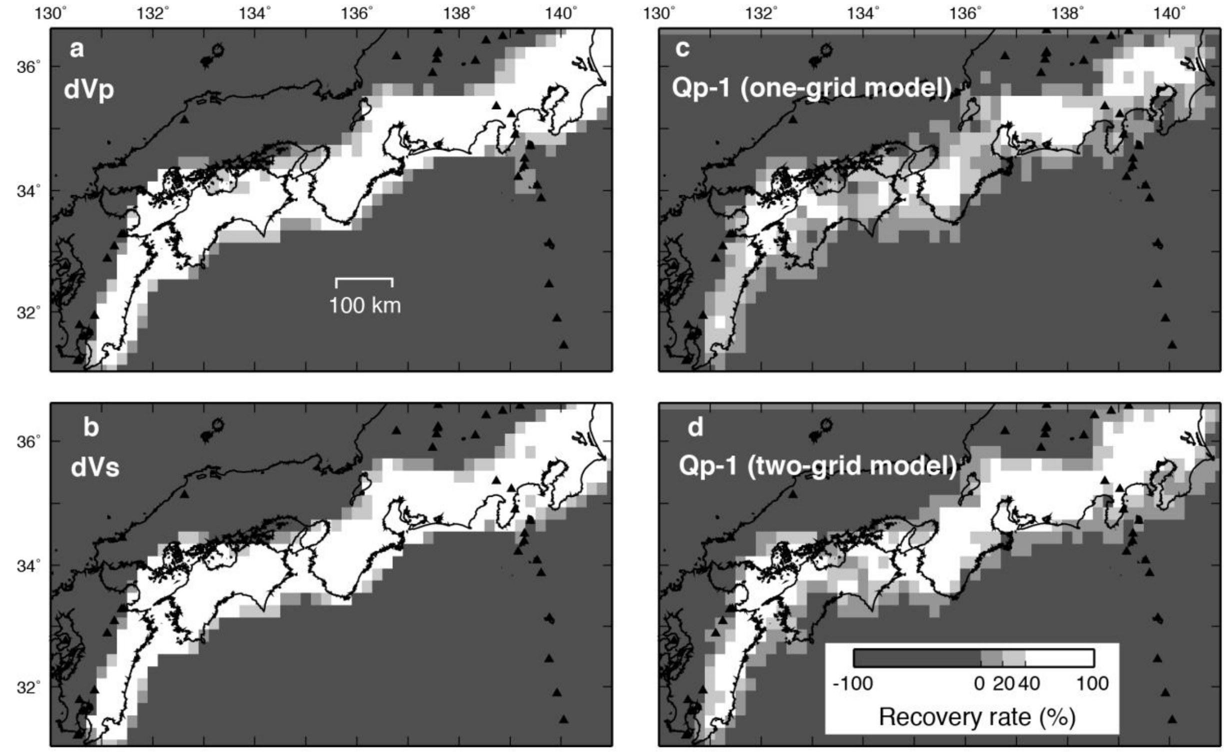

Supplementary Figure 5. Recovery rates of the checkerboard resolution test for **(a)** P-wave and **(b)** S-wave velocities, **(c)** P-wave attenuation for a one-grid model and **(d)** P-wave attenuation for a two-grid model along a curved surface 3 km above the Philippine Sea slab.

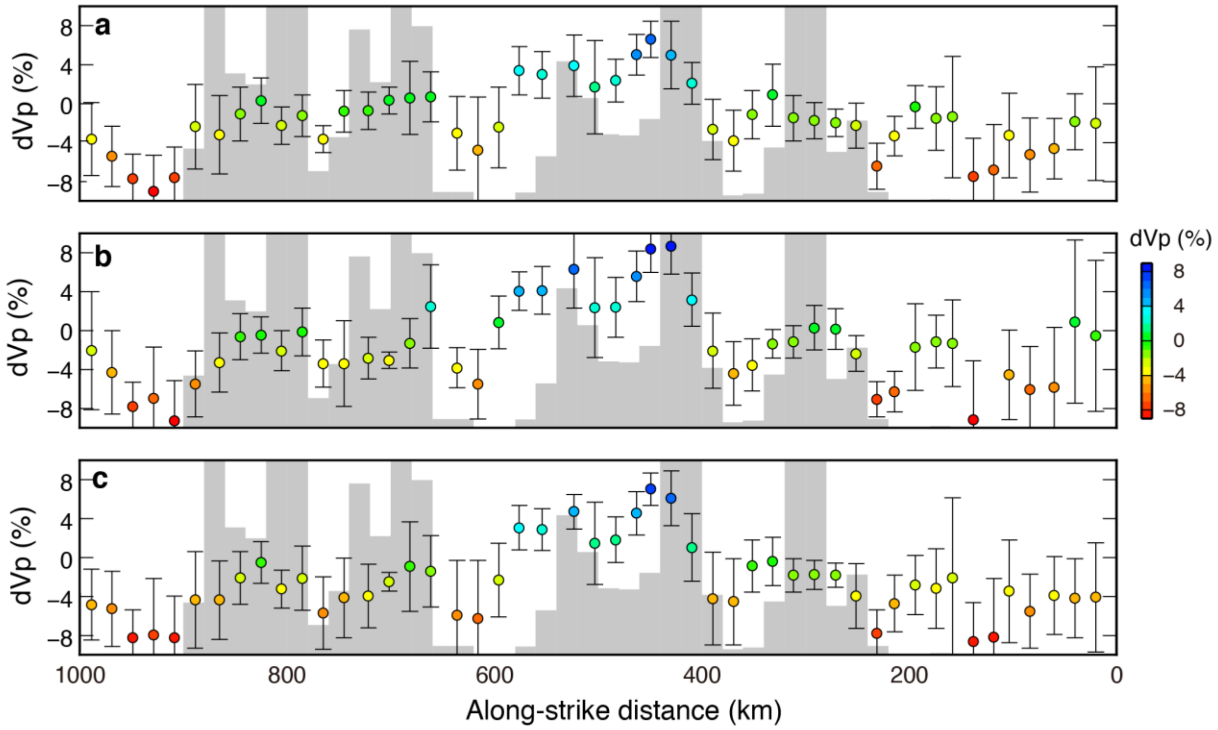

Supplementary Figure 6. Inversion results for the different data sets and model parameterizations. Along-strike variations in P-wave velocity perturbations obtained by inversions with **(a)** non-horizontal geometries of the Conrad and Moho discontinuities[13], **(b)** grid nodes shifted horizontally by a half-grid space, and **(c)** a different data set. The grey histogram in each panel denotes the number of LFEs (the maximum frequency of 1000).

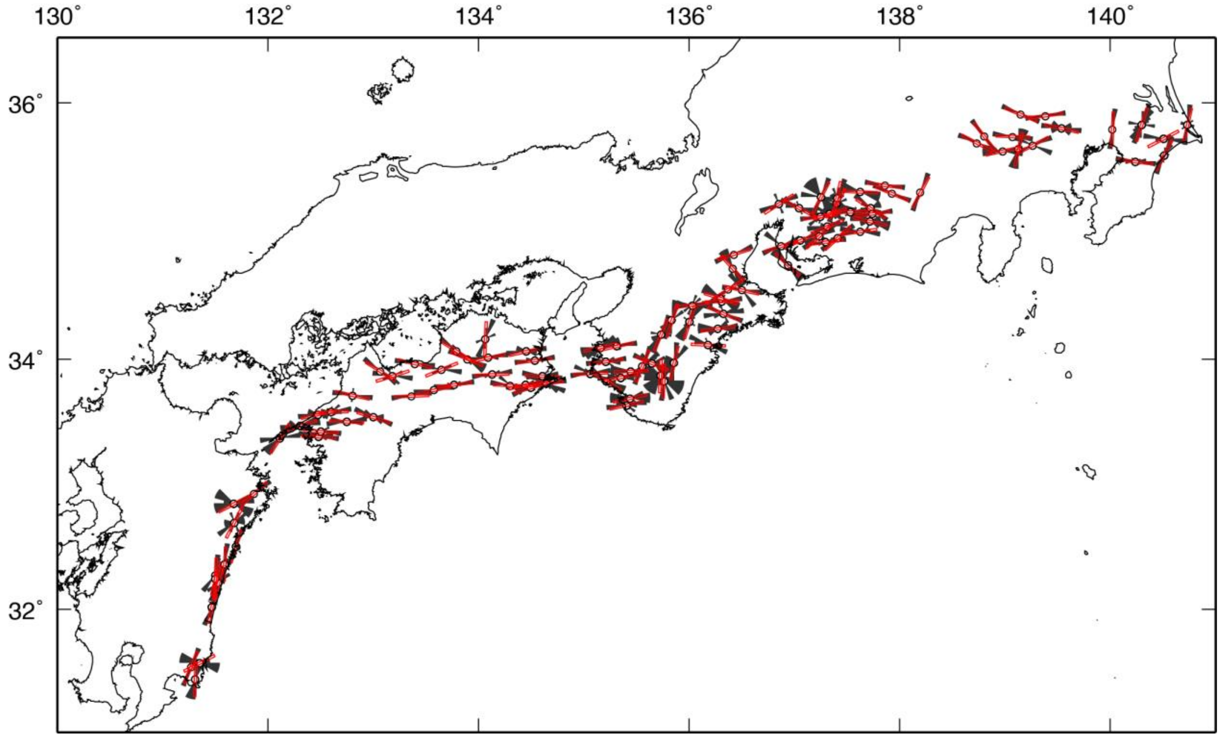

Supplementary Figure 7. Distribution of anisotropy inferred from shear-wave splitting analysis plotted at station locations. Directions of the fast S waves observed for each measurement are shown by the grey rose diagrams, with average directions of anisotropy at each station being denoted by the red lines.

Supplementary References

1. Asano, Y., Obara, K. & Ito, Y. Spatiotemporal distribution of very-low frequency earthquakes in Tokachi-oki near the junction of the Kuril and Japan trenches revealed by using array signal processing. *Earth Planets Space* **60,** 871–875 (2008).
2. Ozawa, S., Suito, H. & Tobita, M. Occurrence of quasi-periodic slow-slip off the east coast of the Boso peninsula, Central Japan. *Earth Planets Space* **59,** 1241–1245 (2007).
3. Fujii, Y. Vertical crustal movement in the Boso peninsula, South Kanto, Japan, as deduced from the adjustment of a geodetic network with signals. *Tectonophysics* **218,** 309–322 (1993).
4. Ohta, Y., Kimata, F. & Sagiya, T. Reexamination of the interplate coupling in the Tokai region, central Japan, based on the GPS data in 1997–2002. *Geophys. Res. Lett.* **31,** L24604 (2004).
5. Kobayashi, A. A long-term slow slip event from 1996 to 1997 in the Kii Channel, Japan. *Earth, Planets Space* **66,** 9 (2014).
6. Kobayashi, A. A small scale long-term slow slip occurred in the western Shikoku in 2005 (in Japanese with English abstract). *Zisin* **63,** 97–100 (2010).
7. Takagi, R., Obara, K. & Maeda, T. Slow slip event within a gap between tremor and locked zones in the Nankai subduction zone. *Geophys. Res. Lett.* **43,** 1066–1074 (2016).
8. Hirose, H., Hirahara, K. & Kimata, F. A slow thrust slip event following the two 1996 Hyuganada Earthquakes beneath the Bungo Channel, southwest Japan. *Geophys. Res. Lett.* **26,** 3237–3240 (1999).
9. Yarai, H. & Ozawa, S. Quasi-periodic slow slip events in the afterslip area of the 1996 Hyuga-nada earthquakes, Japan. *J. Geophys. Res. Solid Earth* **118,** 2512–2527 (2013).
10. Nishimura, T., Matsuzawa, T. & Obara, K. Detection of short-term slow slip events along the Nankai Trough, southwest Japan, using GNSS data. *J. Geophys. Res. Solid Earth* **118,** 3112–3125 (2013).
11. Hirose, F., Nakajima, J. & Hasegawa, A. Three-dimensional seismic velocity structure and configuration of the Philippine Sea slab in southwestern Japan estimated by double-difference tomography. *J. Geophys. Res.* **113,** B09315 (2008).
12. Nakajima, J., Hirose, F. & Hasegawa, A. Seismotectonics beneath the Tokyo metropolitan area, Japan: Effect of slab-slab contact and overlap on seismicity. *J. Geophys. Res.* **114,** B08309 (2009).
13. Katsumata, A. Depth of the Moho discontinuity beneath the Japanese islands estimated

by traveltime analysis. *J. Geophys. Res.* **115,** B04303 (2010).
